# Eustachian tube dysfunction in sleep apnea patients and improvements afforded by continuous positive airway pressure therapy

**DOI:** 10.1016/j.bjorl.2020.02.003

**Published:** 2020-03-12

**Authors:** Serkan Cayir, Omer Hizli, Serkan Kayabasi, Guven Yildirim

**Affiliations:** a Aksaray University, Aksaray Education and Research Hospital, Department of ENT, Aksaray, Turkey; b Giresun University, A. Ilhan Ozdemir Education and Research Hospital, Department of ENT, Giresun, Turkey; c Aksaray University, Faculty of Medicine, Department of ENT, Aksaray, Turkey; d Giresun University, Faculty of Medicine, Department of ENT, Giresun, Turkey

**Keywords:** Sleep apnea, Tympanometry, Middle ear pressure, Continuous positive airway pressure

## Abstract

**Introduction:**

Upper airway resistance may accompany eustachian dysfunction and alter middle ear pressure in patients with obstructive sleep apnea syndrome.

**Objective:**

To investigate effects of obstructive sleep apnea syndrome and continuous positive airway pressure treatment on eustachian tube functions.

**Methods:**

Forty-two mild obstructive sleep apnea syndrome patients, 45 moderate obstructive sleep apnea syndrome patients, 47 severe obstructive sleep apnea syndrome patients with continuous positive airway pressure therapy, 32 severe obstructive sleep apnea syndrome patients without continuous positive airway pressure therapy, and 88 individuals without sleep apnea (controls) were included. Tympanometric parameters of groups were compared.

**Results:**

Right middle ear pressures in mild and moderate obstructive sleep apnea syndrome groups did not differ significantly from that of control group (*p* = 0.93 and *p* = 0.55), as was also true of the left middle ear pressures (*p* = 0.94 and *p* = 0.86). Right middle ear pressure was significantly higher in severe obstructive sleep apnea syndrome groups than in the control group, as was the left middle ear pressure (*p* < 0.001). Middle ear pressure (negative) was significantly lower in severe obstructive sleep apnea syndrome patients with continuous positive airway pressure therapy compared to those without (*p* < 0.001). Right ear type B and C tympanogram frequencies were significantly higher in patients with severe obstructive sleep apnea syndrome without continuous positive airway pressure therapy (12.4%) than in the controls (0%) (*p* = 0.02). Left ear type B or C tympanogram frequencies were significantly higher in patients with severe obstructive sleep apnea syndrome without continuous positive airway pressure therapy (21.9%) than in the controls (0%) (*p* = 0.002).

**Conclusion:**

Mild and moderate obstructive sleep apnea syndrome did not affect middle ear pressure but severe obstructive sleep apnea syndrome may increase the (negative) middle ear pressure. In severe obstructive sleep apnea syndrome patients, long-term continuous positive airway pressure therapy may normalize middle ear pressure.

## Introduction

Obstructive sleep apnea syndrome (OSA), the most common sleep-related disorder, is characterized by recurrent episodes of prolonged partial (hypopnea) and/or complete (apnea) upper airway obstruction. The intermittent hypoxemia and hypercapnia reduce blood oxygen saturation and increase the carbon dioxide concentration.^1^ Such pathological respiratory cycles in OSA patients tend to repeat several times each night; poor-quality sleep is further complicated by snoring and frequent awakening. OSA is associated with comorbid conditions such as cardiovascular illness and chronic obstructive pulmonary disease.^2^, ^3^ Upper airway resistance may be accompanied by Eustachian tube (ET) dysfunction and (negative) middle ear pressure changes in OSA patients. The ET is usually closed, opening only during swallowing, chewing, and sneezing, to balance the middle ear and atmospheric pressures. The ET critically regulates middle ear pressure.^4^ Continuous positive airway pressure (CPAP) application, the gold standard treatment for patients with OSA of all stages, is believed to serve as a pneumatic splint preventing upper airway collapse. It was earlier reported that middle ear pressure was affected by the application of positive pressure.^5^ A rise in the pressure of air passing through the nasopharynx may temporarily increase middle ear pressure via the ET. However, effective OSA treatment via long-term CPAP therapy permanently eliminates the middle ear pressure imbalance, thus removing the upper airway resistance associated with primary OSA. Here, we investigated the association between OSA and ET functions by measuring tympanometric parameters. To explore the effects of long-term CPAP therapy on middle ear pressure, we enrolled patients with severe OSA who had received CPAP treatment for at least 6 months.

## Methods

### Subjects and study design

This study adhered to all relevant principles of the declaration of Helsinki and was approved by the ethics committee (IRB n° 2019-01-07). This was a prospective clinical study; 159 patients admitted to the sleep disorder outpatient clinics of two different institutions were included. All patients gave detailed medical histories and comprehensive otolaryngological and rhinological examinations were performed. All patients underwent whole-of-night type 1 polysomnography (PSG) and tympanometry. The CPAP group was subjected to tympanometric evaluation after at least 6 months of CPAP therapy. We excluded patients with the following conditions that might affect ET function, or reduce tympanic membrane compliance or the middle ear volume: a perforated tympanic membrane, any history of otologic surgery (tympanoplasty and any surgery to treat chronic otitis media), severe septal deviation, nasal polyposis, sinusitis, and/or adenoidal hypertrophy. Patients admitted to the sleep disorder outpatient clinic with complaints of snoring, daytime sleepiness, witnessed apnea, insomnia, and periodic leg movements were included, as were those who underwent PSG because they feared they had OSA (although they had no symptoms). To ensure that our control group did not contain OSA patients, we ensured that all controls exhibited apnea/hypopnea indices (AHIs) less than 5. Additionally, patients with conditions that might affect ET function were not included in the control group.

To explore the effects of OSA and CPAP application on middle ear pressure and the association between OSA severity and ET dysfunction, we formed four study groups by reference to the AHI and CPAP usage status: mild OSA (AHI = 5–14), moderate OSA (AHI = 15–30), severe OSA without CPAP therapy (AHI > 30), and severe OSA with CPAP therapy (initial AHI > 30 and CPAP therapy duration of at least 6 months). Our definitions are those used in previous publications. Mbata et al.^6^ defined OSA as more than five apneas/hypopneas combined with daytime sleepiness, or an AHI > 15. OSA severity is rated using the AHI = 5–14 mild, 15–30 moderate, and > 30 severe. As all patients with AHIs of 5–14 exhibited OSA symptoms, we included them in the mild OSA group. Our control group featured only patients with AHIs < 5.

Tympanometric measurements were performed on both ears of 159 patients in the study group and 88 patients in the control group using an impedance audiometer model AZ 7 (Interacoustics, Assens, Denmark). Tympanograms exhibiting compliance peaks between +50 daPa and −100 daPa were considered type A, those with peaks between −100 daPa and −350 daPa type C, and those with no peaks type B. An Alice 5 Diagnostic Sleep System (Respironics, Murrysville, PA, USA) running Alice Sleepware software was used for whole-of-night PSG. We noted patient age, sex, AHI, and tympanometric parameters, and we compared tympanogram types and middle ear pressures.

### Statistical analysis

Results are presented as median (min‒max). The distribution pattern of the data was investigated using Kolmogorov-Smirnov normality test. Comparison of middle ear pressures among five groups were performed using Kruskal Wallis test. Advanced comparisons of middle ear pressures between the study groups and the control group were performed using Mann Whitney *U* test. The difference of type B and C tympanogram frequencies between the study groups and the control group was investigated using Chi-square test. SPSS 16.0 software for Windows (SPSS Inc., Chicago, IL) was used for statistical analysis. A *p*-value less than 0.05 was considered statistically significant. Additionally for post-hoc comparison tests, Bonferroni correction of five groups (decimal combination) was performed, thus, a *p*-value less than 0.005 (0.05/10) was considered statistically significant.

## Results

In total, 159 patients with OSA (study group) and 88 without OSA (control group) were enrolled. Overall, 42 patients (22 males and 20 females, mean age: 53 ± 9 years) had mild OSA; 45 (27 males and 18 females, mean age: 51 ± 8 years) had moderate OSA; 40 were on CPAP therapy (22 males and 18 females, mean age: 56 ± 8 years) for severe OSA; and 32 were not receiving CPAP therapy (14 males and 18 females, mean age: 55 ± 9 years) for severe OSA. The control group consisted of 88 patients without OSA (42 males and 46 females, mean age: 52 ± 9 years). The distributions of unilaterally and bilaterally affected patients (with type B or C tympanograms) are listed in Table 1. The median right and left middle ear pressures are listed in Table 2. Kruskal-Wallis analysis revealed that both the right and left middle ear pressures differed significantly among the groups (*p* <  0.001). The right middle ear pressures in the mild and moderate OSA groups did not differ significantly from that of the control group (*p*  = 0.93 and *p* = 0.55, respectively), as was also true of the left middle ear pressures (*p* = 0.94 and *p* = 0.86, respectively). In contrast, the right middle ear pressure was significantly higher in both severe OSA groups than in the control group (*p* < 0.001), as was the left middle ear pressure (*p* < 0.001). Thus, severe OSA may increase middle ear pressure. Also, the right and left middle ear pressures were significantly lower in patients with severe OSA receiving CPAP therapy than in those who were not receiving therapy (both *p* < 0.001). CPAP treatment may normalize the middle ear pressure in patients with severe OSA.

**Table 1:** The distributions of unilaterally and bilaterally affected patients in each group.

	Unilaterally affected	Bilaterally affected	Total
Control group	0	0	88
	0%	0%	100%
Mild OSA	0	1	42
	0%	2.4%	100%
Moderate OSA	0	1	45
	0%	2.2%	100%
Severe OSA without CPAP therapy	3	4	32
	9.4%	12.5%	100%
Severe OSA with CPAP therapy	1	2	40
	2.5%	5%	100%
Total	4	8	247
	1.6%	3.2%	100%

**Table 2:** The median right and left middle ear pressures (dPa).

Groups	Right ears	Left ears
Control group	−12.5 [(-48.22)‒(18.22)]	−12.44 [(−44.14)‒(18.57)]
Mild OSA	−12.68 [(-42.16)‒(8.12)]	−12.64 [(−43.74)‒(9.41)]
Moderate OSA	−12.14 [(-37.82)‒(6.41)]	−12.28 [(−35.17)‒(7.14)]
Severe OSA without CPAP therapy	−68.98 [(-123.59)‒(-31.48)]	−68.52 [(−136.41)‒( −32.01)]
Severe OSA with CPAP therapy	−34.7 [(-112.86)‒(-12.28)]	−32.58 [(−115.67)‒( −11.43)]
*p*-value^a^	< 0.001	< 0.001

OSA, Obstructive Sleep Apnea Syndrome; CPAP, Continuous Positive Airway Pressure.

a Kruskal Wallis analysis.

Table 3, Table 4 list the tympanogram type distributions of the right and left ears, respectively. The right-ear type B and C tympanogram frequencies were significantly higher in patients with severe OSA not receiving CPAP therapy (12.4%) than in the controls (0%) (X^2^ = 5.4 and *p* = 0.02). The frequencies of left-ear type B or C tympanograms were significantly higher in patients with severe OSA not receiving CPAP therapy (21.9%) than in the controls (0%) (X^2^ = 9.9 and *p* = 0.002). However, the frequencies of right-ear type B or C tympanograms did not differ significantly between patients with severe OSA receiving CPAP therapy (5%) and the controls (0%) (X ^2^ = 2.1 and p = 0.14). Furthermore, the frequencies of left-ear type B or C tympanograms did not differ significantly between patients with severe OSA receiving CPAP therapy (7.5%) and the controls (0%) (X^2^ = 3.1 and *p* = 0.07). Thus, CPAP therapy may reduce the frequencies of type B or C tympanograms in patients with severe OSA.

**Table 3:** Tympanogram type distributions of the right ears.

	Right ears			Total
	Type A	Type B	Type C	
Control group	88	0	0	88
	100%	0%	0%	100%
Mild OSA	41	1	0	42
	97.6%	2.4%	0%	100%
Moderate OSA	44	1	0	45
	97.8%	2.2%	0%	100%
Severe OSA without CPAP therapy	28	2	2	32
	87.5%	6.2%	6.2%	100%
Severe OSA with CPAP therapy	38	1	1	40
	9.5%	2.5%	2.5%	100%
Total	239	5	3	247
	96.8%	2%	1.2%	100%

**Table 4:** Tympanogram type distributions of the left ears.

	Left ears			Total
	Type A	Type B	Type C	
Control group	88	0	0	88
	100%	0%	0%	100%
Mild OSA	41	1	0	42
	97.6%	2.4%	0%	100%
Moderate OSA	44	1	0	45
	97.8%	2.2%	0%	100%
Severe OSA without CPAP therapy	25	4	3	32
	78.1%	12.5%	9.4%	100%
Severe OSA with CPAP therapy	37	2	1	40
	92.5%	5%	2.5%	100%
Total	235	8	4	247
	95.1%	3.2%	1.6%	100%

## Discussion

OSA is a significant problem, being common and associated with comorbid diseases and complications. OSA negatively impacts the middle and inner ears.^7^, ^8^, ^9^ The upper airway resistance that causes snoring and breathing cessation in OSA patients may reflect increased (negative) middle ear pressure. CPAP therapy may normalize middle ear pressure. Thus, we explored ET function with tympanography in OSA patients. Unlike previous publications,^10^ we included patients with mild, moderate, and severe OSA, and we evaluated the association between OSA severity and ET function. Severe OSA patients were divided into two groups by CPAP therapy status to explore the long-term effects of such therapy. Mild and moderate OSA did not affect middle ear pressure. However, severe OSA was significantly associated with increased (negative) middle ear pressure, indicating impaired ET functionality. Also, CPAP therapy at least 6 months in duration significantly decreased the (negative) middle ear pressure. This normalizing effect might reflect the positive pressure imparted by air passing through the nasopharynx. We hypothesize that long-term CPAP therapy permanently balances middle ear pressure by eradicating upper airway resistance.

The ET controls air pressure equilibration, middle ear ventilation, mucociliary clearance of middle ear secretions, and middle ear protection. Middle ear pressure is controlled by mucosal gas exchange between the middle ear and the ET opening; the pressure becomes that within the nasopharynx.^11^ The relative contributions of the various pressure-regulating systems to normal middle ear ventilation remain unclear. As the ET is normally closed, the pressure within a healthy middle ear gradually decreases. Periodic ET opening allows air at atmospheric pressure to enter, balancing the middle ear pressure.^12^ The ET normally clears middle ear secretions and protects the middle ear from inflammation and infection. We excluded patients with conditions that might affect ET function. The literature does not describe the tympanogram type distributions of OSA patients. Our data are presented in Table 2, Table 3, evidencing an effect of OSA on ET function. The type B/C tympanogram frequencies were significantly higher in patients with severe OSA who did not receive CPAP therapy than in controls. However, the type B/C tympanogram frequencies did not differ significantly between the patients with severe OSA receiving CPAP therapy and controls. Thus, severe OSA may significantly increase (negative) middle ear pressure, associated with type B/C tympanograms, but long-term CPAP treatment may reduce the frequencies of such tympanograms.

CPAP treatment side effects include nasal congestion and dryness, conjunctivitis, epistaxis, otalgia, mask-associated discomfort, skin irritation, and mouth dryness. However, CPAP therapy effectively treats OSA.^13^, ^14^ Meslier et al.^15^ found that CPAP therapy was employed by 76% of 3225 patients. Long-term CPAP treatment improves the quality of life and restores ET function in patients with severe OSA.

Few data on the effects of OSA severity on ET function are available in the English-language literature. We found that mild and moderate OSA did not affect middle ear ventilation. Tympanometric evaluation may be appropriate during follow-up for patients with severe OSA. This prospective clinical study revealed a cross-sectional association between ET dysfunction and OSA status. However, we cannot explain why ET function is impaired in OSA patients. Further work is required.

## Conclusion

Mild and moderate OSA did not affect middle ear pressure, but severe OSA may increase the (negative) middle ear pressure. In severe OSA patients, long-term CPAP therapy may enhance ET function and normalize middle ear pressure. Familiarity with the effects of OSA and CPAP treatment on the upper respiratory tract and ET will improve the management of patients with severe OSA and pathology of the middle ear.

## Conflicts of interest

The authors declare no conflicts of interest.
